# Development and Validation of Stability Indicating Spectroscopic Method for Content Analysis of Ceftriaxone Sodium in Pharmaceuticals

**DOI:** 10.1155/2014/278173

**Published:** 2014-08-24

**Authors:** Revathi Ethiraj, Ethiraj Thiruvengadam, Venkattapuram Saravanan Sampath, Abdul Vahid, Jithin Raj

**Affiliations:** ^1^Department of Pharmaceutical Analysis, The Erode College of Pharmacy, Erode, Tamil Nadu 638112, India; ^2^Department of Pharmaceutics, The Erode College of Pharmacy, Erode, Tamil Nadu 638112, India

## Abstract

A simple, selective, and stability indicating spectroscopic method has been selected and validated for the assay of ceftriaxone sodium in the powder for injection dosage forms. Proposed method is based on the measurement of absorbance of ceftriaxone sodium in aqueous medium at 241 nm. The method obeys Beer's law in the range of 5–50 *μ*g/mL with correlation coefficient of 0.9983. Apparent molar absorptivity and Sandell's sensitivity were found to be 2.046 × 10^3^ L mol^−1^ cm^−1^ and 0.02732 *μ*g/cm^2^/0.001 absorbance units. This study indicated that ceftriaxone sodium was degraded in acid medium and also underwent oxidative degradation. Percent relative standard deviation associated with all the validation parameters was less than 2, showing compliance with acceptance criteria of Q2 (R1), International Conference on Harmonization (2005) guidelines. Then the proposed method was successfully applied to the determination of ceftriaxone sodium in sterile preparation and results were comparable with reported methods.

## 1. Introduction

**Figure 1:**
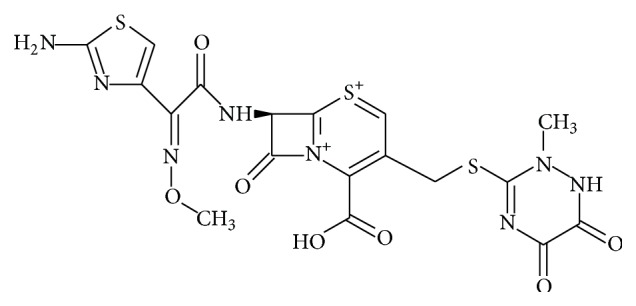
Chemical structure of ceftriaxone sodium.

Ceftriaxone sodium (Figure 1) is chemically (6*R*,7*R*)-7-[[(2*Z*)-(2-amino-4-thiazolyl)(methoxyimino)acetyl]amino]-8-oxo-3-[[(1,2,5,6-tetrahydro-2-methyl-5,6-ioxo-1,2,4-triazin-3-yl)thio]-methyl]-5-thia-1-azabicyclo[4.2.0]oct-2-ene-2-carboxylic acid [1]. Ceftriaxone is a third generation, semisynthetic cephalosporin antibiotic. Cephalosporins are derivatives of 7-aminocephalosporic acid and are closely related to penicillins in structure. Ceftriaxone sodium is a long acting, broad-spectrum cephalosporin antibiotic for parenteral use. The bactericidal activity of ceftriaxone sodium results from inhibition of cell wall synthesis. It exerts in vitro activity against a wide range of Gram-negative and Gram-positive microorganisms. It is highly stable to most beta-lactamases, both penicillinases and cephalosporinases, of Gram-positive and Gram-negative bacteria.

A thorough literature survey has revealed that HPLC method for ceftriaxone sodium with combination of other drugs and individually in dosage forms [2–15], microbial bioassay methods [16, 17], and spectrophotometric methods in dosage forms [18–22] have been reported for analysis of ceftriaxone sodium. Costlier and volatile solvents were used as mobile phase solvent system and the time of analysis was more in some reported methods.

Some spectrophotometric methods are recently described in the literature for analysis of drugs in raw material and finished products such as ceftazidime [23–25], cefuroxime [26], and cefazolin [27]. These spectrophotometric methods involve the use of no toxic organic solvents, which do not contribute to the generation of this kind of waste by the chemicals or industries. In this context, spectrophotometry stands out. Therefore, the trend is that the industries look for ways to reduce the impacts of their activities on the environment. So, the principle objective of this study was, therefore, to develop a simple, selective, precise, less time consuming, and economical method with a wide linear range and good sensitivity for assay of ceftriaxone sodium in the powder for injection dosage forms.

The parent drug stability guidelines issued by the international conference of harmonization (ICH) [28] require that analytical test procedure should indicate stability. Therefore, the present study was extended to establish the inherent stability of ceftriaxone sodium under different stress conditions such as, alkaline, acidic, oxidative, and photolytic conditions. Thus, this method can be utilized to compare the results for the content analysis of stability samples, since the purpose of stability studies is to monitor possible changes to a product or a material over a time at different storage conditions.

## 2. Materials and Methods

### 2.1. Instruments and Chemicals

Spectrophotometric analysis was carried out on a Systronics 2201, UV-visible double-beam spectrophotometer with matched 1 cm path-length quartz cells. Absorption spectra were recorded on a medium scan speed, setting slit width to be 1 nm. Ceftriaxone sodium was obtained as a gift sample from Orchid Chemicals and Pharmaceutical Ltd., Chennai, India. The other chemicals like sodium hydroxide, hydrochloric acid, and hydrogen peroxide used were of Merck-AR grade and double distilled water was used throughout the experiment.

### 2.2. Preparation of Standard Stock Solution

A stock solution was prepared by dissolving 100 mg of ceftriaxone sodium pure drug in 100 mL of distilled water to get standard stock solution (1 mg/mL).

### 2.3. Construction of the Calibration Curve

To a set of 10 mL volumetric flasks, aliquot volumes containing the drug were quantitatively transferred from standard stock solution and made up to the mark with distilled water to obtain final concentrations of 5–50 *μ*g/mL of ceftriaxone sodium. The absorbance was measured at 241 nm and calibration graph was constructed by plotting the absorbance versus the concentration of the drug. Alternatively, the corresponding regression equation was derived.

### 2.4. Validation of the Proposed Method

The validity of the method was tested regarding linearity, specificity, accuracy, and precision according to ICH Q2B recommendations.

#### 2.4.1. Accuracy and Precision

Accuracy and precision of the method were evaluated with the help of percent recovery, standard deviation (SD), and percent relative standard deviation (RSD) by using standard addition method. Three levels of standard drug (10%, 20%, and 30%) were spiked individually with the 100 mg equivalent of powder for injection dosage form ceftriaxone sodium and analysed in six replicates during the same day (intraday precision) and six consecutive days (interday precision). The results obtained were tabulated along with standard error and 95% confidence interval.

#### 2.4.2. Linearity

A linear correlation was found between absorbance at *λ* max and various concentrations of ceftriaxone sodium. The linearity graph obeyed Beer's law in the range from 5 to 50 *μ*g/mL and it was described by regression equation (*y* = *mx* + *c*) and correlation coefficient (*r* ^2^) which were displayed on the graph. Molar absorptivity, Sandell's sensitivity, standard error on slope, confidence limit of slope (95%), standard error on intercept, confidence limit of intercept (95%), LOD, and LOQ were calculated.

#### 2.4.3. Limit of Detection (LOD) and Limit of Quantification (LOQ)

For the determination of LOD and LOQ, the method is based on residual standard deviation of regression line and slope. To determine LOD and LOQ, the specific calibration curve was studied using the sample containing analyte in the range of detection limit and quantitation limit.

#### 2.4.4. Robustness

Robustness was studied by evaluating the influence of small but deliberate variations in the experimental condition like storing the similar concentration (20 *μ*g/mL) of drug solution at two different temperatures (20°C and 30°C) for 1 h and performing the stability of the sample solution at various time intervals (after 1 h and after 6 h) on the analytical performance.

#### 2.4.5. Forced Degradation Study

A 2 mL aliquot of standard stock solution of ceftriaxone sodium (1 mg/mL) was taken in four replicates in a volumetric flask (100 mL) and mixed with 10 mL of 0.1 N HCl (acid hydrolysis) or 0.1 N NaOH (alkaline hydrolysis) or 5% H_2_O_2_ (oxidative degradation) and set aside for 1 h at room temperature. Solution was diluted up to mark with distilled water. For photolytic degradation, a solution of drug (20 *μ*g/mL) was prepared as per the procedure under construction of the calibration graph and was exposed to UV radiation of wavelength 254 nm and of 1.4 flux intensity for 24 h in a UV chamber. For thermal degradation solid drug was kept in an oven at 100°C for 24 h. After cooling to room temperature, 20 *μ*g/mL concentrated drug solution was prepared as per above said method. Finally, absorbance of all the solutions resulted from acid and alkaline hydrolysis, and oxidative degradation, photolytic degradation, and thermal degradation were measured at 241 nm against respective solvent as blank in each case.

### 2.5. Application of Developed Method to Analyse Drug Formulation

For the estimation ceftriaxone sodium from injection (by procuring three brands from market), a portion of powder equivalent to 100 *μ*g of the drug from each brand was accurately weighed and transferred into 100 mL volumetric flask. The drug was dissolved by adding 70 mL of distilled water and sonicated for 15 min. The volume was completed with distilled water and filtered. Aliquot containing suitable concentration (20 *μ*g/mL) of ceftriaxone sodium was analyzed as described under construction of the calibration graph. The nominal content of the drug in injection for each brand was determined using the corresponding regression equation and results of %RSD of drug content were statistically compared with reported method.

## 3. Results and Discussion

**Figure 2:**
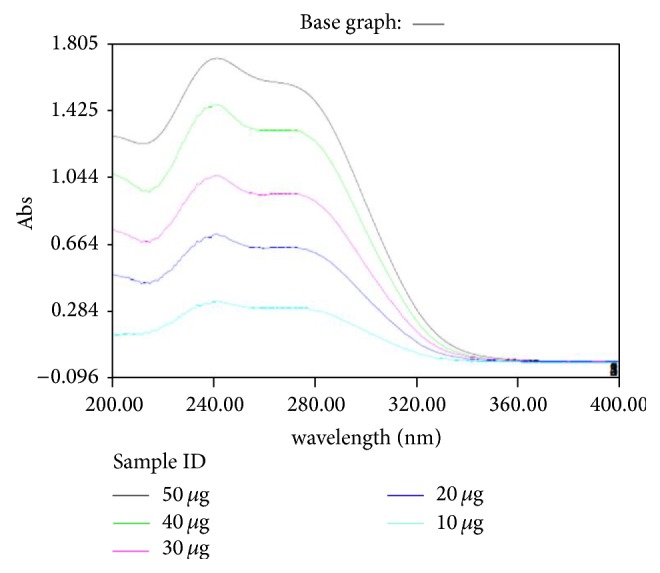
Overlay spectrum for ceftriaxone sodium (*λ* max at 241 nm).

Aqueous solution of ceftriaxone sodium showed absorbance maximum (*λ* max) at 241 nm (Figure 2), and at this wavelength distilled water did not show any significant absorbance. Therefore further analysis was carried out at 241 nm.

**Figure 3:**
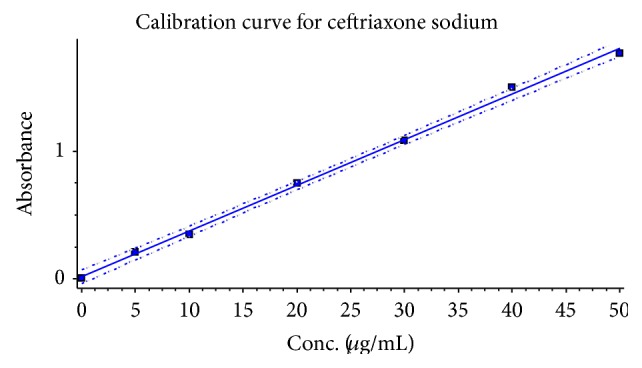
Calibration curve for ceftriaxone sodium.

Least square regression equation of ceftriaxone sodium in aqueous medium has shown that the *R* ^2^ value very close to 1 indicated high degree of correlation between two variables, such as absorbance and concentration (Figure 3). Beer's law was obeyed over the range of 5–50 *μ*g/mL; high values of molar absorptivity and low values of Sandell's sensitivity and LOD revealed that proposed methods are highly sensitive. LOD and LOQ values for the drug were found and all the parameters of calibraration curve were displayed in Table 1.

To check accuracy and precision, assays were carried out for six times within a day (intraday precision) and in six consecutive days (interday precision) by adding three different levels of analyte to the formulation. % RSD values were ≤0.5 (intraday) and ≤1.18 (interday) indicating high precision of developed method. Accuracy of the method was ascertained as mean % recovery between measured actual concentration and taken concentration for ceftriaxone sodium. The values of % recovery, very close to 100%, demonstrate high accuracy of the proposed method (Table 2).

Robustness studies assumed that the small variations in any of the variables did not significantly affect the results (Table 3). This provided an indication for the reliability of the proposed method during routine analysis.

Ceftriaxone sodium sterile formulations were analysed by proposed method and by reported method [21], which involved the analysis of analyte with 0.1 M sodium hydroxide and absorbance measured at 258.8 nm. Results were compared by Student's* t*-test and variance-ratio *F*-test. Calculated *t*-values and *F*-values did not exceed tabulated values of 2.776 (*t*) and 6.39 (*F*) at 95% confidence level and for four degrees of freedom (Table 4) which indicates close similarity between proposed and reported method.

Stability indicating property of analyte was performed by forced degradation studies. Ceftriaxone sodium was subjected to various stress conditions like acid, alkaline, hydrogen peroxide induced degradation, and thermal and photolytic condition. Analysis was performed by measuring absorbance of ceftriaxone sodium after subjecting it to stressed conditions at *λ* max of pure drug. Percentage degradation was calculated by the formula: % degradation = (expected concentration−actual concentration)/expected concentration × 100, and percentage recovery also was calculated for each case (Table 5). Results revealed no change in absorbance of drug solution at alkaline hydrolysis, so its percentage recovery is very close to 100% which indicates the drug stability. The analyte showed slight degradation with UV and thermal stressed condition. But there was significant change in absorbance after acid and hydrogen peroxide treatment, confirming that ceftriaxone sodium is susceptible to acid hydrolysis (% degradation of 11.0%) and oxidation (% degradation of 7.95%).

## 4. Conclusion

Ceftriaxone sodium was subjected to various studies as per ICH guidelines. Analyte undergoes significant degradation under acid hydrolysis and oxidation, whereas it is stable under alkaline treatment. Proposed method was validated for linearity, accuracy, precision, and robustness and method was applied to various formulations and results were statistically compared with reference method which showed that there were no significant changes in the result. So, method can be utilized for determination of purity of drug available from various sources without any tedious procedure and also used in analysis of stability study samples.

## Figures and Tables

**Table 1:** Linearity data of developed method.

Parameters	Values
*λ* max (nm)	241
Linearity range (*μ*g/mL)	5–50
LOD (*μ*g/mL)	0.0332
LOQ (*μ*g/mL)	0.1008
Molar absorptivity (L mol^−1^cm^−1^)	2.046 × 10^3^
Sandell's sensitivity (*μ*g/cm^2^/0.001 absorbance unit)	0.02732
Slope	0.03576
Standard error on slope	0.0007346
Confidence limit of slope (95%)	0.03387–0.03765
Intercept	0.01246
Standard error on intercept	0.02064
Confidence limit of intercept (95%)	0.04060–0.06551
Correlation coefficient	0.9979
Standard deviation of residuals	0.03360

**Table 2:** Interday and intraday accuracy and precision study data.

Level of standard drug added	Interday				Intraday			
	Mean % recovery∗	% RSD∗	S.E	95% CI	Mean % recovery∗	% RSD∗	S.E	95% CI
10%	101.10	0.69	0.4041	99.36–102.84	100.27	0.50	0.2906	99.01–101.52
20%	100.97	1.18	0.6984	99.60–101.90	99.23	0.20	0.1202	98.71–99.75
30%	100.43	1.10	0.6386	97.68–103.18	100.6	0.39	0.2309	99.60–101.59

^*^(*n* = 6); % RSD: percentage relative standard deviation; S.E: standard error; 95% CI: 95 percent confidence interval.

**Table 3:** Data for robustness of the method.

Parameters	Mean % content	% RSD
Temperature		
at 20°C	99.72	1.1055
at 30°C	100.49	1.7369
Time		
after 1 hr	101.74	1.0835
after 6 hr	100.23	1.7414

**Table 4:** Statistical comparison of proposed method with reference method.

Brand name	% RSD for % content∗		Statistical data	
	Proposed method	Reference method	Variance-ratio *F*-test	Student's *t*-test
Cefaxone	0.893	0.672	1.748	0.8819
Cadiceft	0.435	0.555	1.574	2.215
Monocef	1.057	0.468	5.098	0.1255

(^*^ *n* = 3).

**Table 5:** Results for stability of drug under forced degradation study.

Parameters studied	Conc. taken (*μ*g/mL)	Conc. found (*μ*g/mL)	% degradation	% recovery
Acid hydrolysis	20	7.80	11.0	89.0%
Alkaline hydrolysis	20	20.07	−0.35	100.35%
Oxidative degradation	20	18.41	7.95	92.05%
UV degradation	20	21.69	−8.45	108.45%
Thermal degradation	20	19.31	3.45	96.55%
